# Risk and prognostic factors of breast cancer with liver metastases

**DOI:** 10.1186/s12885-021-07968-5

**Published:** 2021-03-06

**Authors:** Lei Ji, Lei Cheng, Xiuzhi Zhu, Yu Gao, Lei Fan, Zhonghua Wang

**Affiliations:** 1 Department of Medical Oncology, Fudan University Shanghai Cancer Center, Shanghai, China; 2 Department of Oncology, Shanghai Medical College, Fudan University, Shanghai, China; 3 Department of Breast Surgery, Fudan University Shanghai Cancer Center, Shanghai, China

**Keywords:** Breast cancer, Liver metastasis, Risk factors, Prognostic factors, HER2-targeted therapy

## Abstract

**Background:**

Liver metastasis is a significant adverse predictor of overall survival (OS) among breast cancer patients. The purpose of this study was to determine the risk and prognostic factors of breast cancer with liver metastases (BCLM).

**Methods:**

Data on 311,573 breast cancer patients from the Surveillance, Epidemiology, and End Results (SEER) database and 1728 BCLM patients from Fudan University Shanghai Cancer Center (FUSCC) were included. Logistic regression was used to identify risk factors for liver metastasis. Cox proportional hazards regression model was adopted to determine independent prognostic factors in BCLM patients.

**Results:**

Young age, invasive ductal carcinoma, higher pathological grade, and subtype of triple-negative and human epidermal growth factor receptor 2 positive (HER2+) were risk factors for developing liver metastasis. The median OS after liver metastasis was 20.0 months in the SEER database and 27.3 months in the FUSCC dataset. Molecular subtypes also played a critical role in the survival of BCLM patients. We observed that hormone receptor-positive (HR+)/HER2+ patients had the longest median OS (38.0 for SEER vs. 34.0 months for FUSCC), whereas triple-negative breast cancer had the shortest OS (9.0 vs. 15.6 months) in both SEER and FUSCC. According to the results from the FUSCC, the subtype of HR+/HER2+ (hazard ratio (HR) = 2.62; 95% confidence interval (CI) = 1.88–3.66; *P* < 0.001) and HR−/HER2+ (HR = 3.43; 95% CI = 2.28–5.15; *P* < 0.001) were associated with a significantly increased death risk in comparison with HR+/HER2- patients if these patients did not receive HER2-targeted therapy. For those who underwent HER2-targeted therapy, however, HR+/HER2+ subtype reduced death risk compared with HR+/HER2- subtype (HR = 0.74; 95% CI = 0.58–0.95; *P* < 0.001).

**Conclusions:**

Breast cancer patients at a high risk for developing liver metastasis deserve more attention during the follow-up. BCLM patients with HR+/HER2+ subtype displayed the longest median survival than HR+/HER2- and triple-negative patients due to the introduction of HER2-targeted therapy and therefore it should be recommended for HER2+ BCLM patients.

**Supplementary Information:**

The online version contains supplementary material available at 10.1186/s12885-021-07968-5.

## Background

Breast cancer is the most frequently diagnosed cancer and is the second leading cause of cancer-related death among women in developed countries [1]. More than 5% of cases are metastatic disease at the time of diagnosis, while almost 30% of patients newly diagnosed with localized or regional disease will recur [2, 3]. Early breast cancer patients has a 5-year survival rate of more than 90%, while it sharply decline to 26% for those with de novo metastatic breast cancer (MBC) [2]. Notably, the incidence of liver metastasis is only second to bone and lung metastasis, accounting for 71% of all patients in an autopsy study [4, 5]. Moreover, liver metastasis could result in treatment resistance and higher mortality. Median survival for breast cancer with liver metastases (BCLM) was only 3–15 months, with a 5-year survival rate of only 8.5% [6, 7]. Because liver metastasis is an important factor influencing long-term survival of breast cancer patients, early identification may offer an opportunity for curative hepatic resection and prolonging the survival time [8]. However, routine screening for distant metastasis among patients without clinical signs and symptoms related to relapse is not recommended according to breast cancer follow-up or surveillance guidelines due to lack of demonstrated clinical benefits [9]. Thus, BCLM patients who usually present asymptomatically or with atypical symptoms tend to be ignored in the beginning stage of liver metastasis.

Several clinicopathological factors, especially molecular subtypes, can influence the occurrence and prognosis of liver metastasis. A study which included 3726 early breast cancer patients diagnosed from 1986 to 1992 showed that luminal/HER2 and HER2-enriched tumors were more susceptible to brain, liver, and lung metastases than luminal A tumors [10]. Dent et al. found that triple negative breast cancer had a higher risk of a visceral metastasis compared with other breast cancer subtypes [11]. Importantly, molecular subtypes are not only a risk factor for liver metastasis but also a predictor of clinical outcome of BCLM patients. A registry analysis of 500 BCLM patients found HR positive breast cancer reduced the risk of death by 33% compared with HR negative breast cancer [7]. A retrospective study of 145 BCLM patients also showed that estrogen receptor (ER) positive patients had a longer median than ER negative patients (7 vs 3.65 months) [12]. Additionally, multiple studies have shown that triple negative breast cancer has the worst prognosis among BCLM patients [13, 14]. There are some controversial or even contradicting results, however, regarding the prognostic value of molecular subtypes in BCLM patients. A previous study including 104 BCLM patients concluded that there was no impact of breast cancer subtypes on the survival after hepatic metastases [15]. A single center study of British population also presented similar results [16]. HER2 positive breast cancer is associated with a more aggressive phenotype and worse prognosis. In an earlier study, Kennecke et al. reported that MBC patients with luminal A subtype had longer survival than those with luminal/HER2 subtype (2.2 vs 1.3 years) owing to the lack of effective targeted therapy [10]. However, HER2 positive MBC patients with the addition of trastuzumab had better prognosis than those with HER2 negative disease historically considered to have a relatively favorable prognosis [17]. A recent population-based study on de novo BCLM patients also observed that patients with HR+/HER2+ subtype exhibited the longest median survival time of 31 months, substantially better than those with HR+/HER2- subtype (21 months) and other subtypes [14].

These above studies including a relatively small sample size are largely based on retrospective data and performed at a single academic center, yielding these different or even contradicting results. Our study aimed to explore risk factors for liver metastases (LM) to identify breast cancer patients at a high risk of liver metastasis at initial diagnosis. In addition, we hoped to detect independent prognostic factors in BCLM patients based on population-based data from the SEER database and large sample size data from the FUSCC dataset. As a result, we were able to evaluate the consistency and difference of these prognostic factors in two different populations and investigate the reasons behind.

## Methods

Within the SEER Research Data 1975–2016 dataset, breast cancer patients diagnosed from 2010 to 2016 were extracted [18]. Inclusion criteria were as follows: (a) age ≥ 18 years; (b) histologically confirmed diseases; (c) known liver metastases status. The exclusion criteria were as follows: (a) carcinoma in situ; (b) multiple primary malignant tumors; (c) unknown follow-up or survival months of 0 month, including patients diagnosed via autopsy or a death certificate. Finally, there were 311,573 breast cancer patients for further analysis, among whom there were 15,884 patients with de novo MBC at initial diagnosis (Fig. S1). Within the FUSCC dataset collected between 2007 and 2018, there were a total of 3453 female MBC patients. The diagnosis of liver metastases was based on the radiologic scan, the biopsy of metastatic lesions or the surgical resection specimens. Inclusion criteria were as follows: (a) age > 20 years; (b) histologically confirmed diseases; (c) developing liver metastases during the course of the disease; (d) detailed medical records. The exclusion criteria were as follows: (a) carcinoma in situ; (b) patients with bilateral breast cancer or other malignant diseases; (c) unknown follow-up. Ultimately, 1728 BCLM patients were eligible for subsequent analysis (Fig. S2). Among 626 HER2 positive BCLM patients, 528 patients with subsequent HER2-targeted therapy information were enrolled to explore the effect of HER2-targeted therapy. Last follow-up was conducted on June 15, 2019, and the median follow-up time was 17.4 months (interquartile range [IQR], 8.5 to 31.0 months).

Incidence was defined as the number of BCLM patients divided by the total number of breast cancer patients or MBC patients in the SEER database. First liver metastases referred to liver metastases as the initial metastatic site, while subsequent liver metastases suggested that breast cancer cells gradually metastasized to the liver in the development of disease. Breast cancer subtypes were categorized as follows: HR-positive/HER2-negative (HR+/HER2-); HR+/HER2+; HR−/HER2+; HR−/HER2- (triple-negative) and unknown. Variables in this study included demographic characteristics and clinicopathological factors such as histology, pathological grade, number of extrahepatic metastatic sites, treatment information and subtype. OS was calculated from the date of diagnosis of liver metastases to the date of death from any cause, and patients alive at the date of last follow-up were censored.

Multivariate logistic regression analyses were performed to determine the risk factors associated with the presence of liver metastases at initial diagnosis. Odds ratios (ORs) with 95% confidence intervals (CIs) were also calculated. Univariate and multivariate Cox regression analyses were adopted to identify the prognostic factors associated with increased all-cause mortality. We also calculated HRs and 95% CIs in the Cox regression model. The consistency and difference of these prognostic factors in two different populations was evaluated on the basis of the data from the SEER database and the FUSCC dataset, and then we attempted to explore the possible causes behind. The Kaplan–Meier method and a log-rank test were used to estimate survival and evaluate differences between survival curves. Statistical analyses were performed using R (version 3.5.1), and a two-sided *P*-value less than 0.05 was considered statistically significant.

## Results

### Baseline characteristics and the incidence of liver metastases

A total of 311,573 patients from SEER database diagnosed between 2010 and 2016 were included in the present study. Of these patients, there were 15,884 MBC patients and 4067 BCLM patients at initial diagnosis (Table 1). In the FUSCC dataset, there were 1728 of 3048 metastatic breast cancer patients had liver metastases during the follow-up (Table 2). The consistent and inconsistent characteristics of two datasets were shown in Tables 1 and 2. The baseline characteristics showed a higher proportion of patients with infiltrating duct carcinoma (73.1% vs 80.8%), extrahepatic metastases (73.9% vs 67.5%) and HR+/HER2− subtype (39.6 44.3%) in the both SEER and FUSCC dataset. However, the differences between SEER and FUSCC were significant as well, such age, race, stage at initial diagnosis. The patients in the FUSCC dataset were younger and almost all of them were Asian patients, different from those in the SERR database. Most notably, the majority of FUSCC patients (88.3%) were recurrent breast cancer who underwent curative resection for primary tumors while all patients from the SEER database were diagnosed with de novo metastatic breast cancer.

**Table 1:** Baseline characteristics of breast cancer with liver metastases at diagnosis in the SEER database

Variable	Patients, No. %	
	Total (*n* = 311,573)	With Liver Metastases (*n* = 4067)
Age, y		
18–40	21,313 (6.8%)	494 (12.1%)
41–60	137,299 (44.1%)	1882 (46.3%)
61–80	129,497 (41.6%)	1414 (34.8%)
> 80	23,464 (7.5%)	277 (6.8%)
Race		
White	207,400 (66.6%)	2565 (63.1%)
Black	35,062 (11.3%)	737 (18.1%)
Hispanic	37,499 (12.0%)	427 (10.5%)
Asian or Pacific Islander	27,997 (9.0%)	305 (7.5%)
American Indian/Alaska Native	1757 (0.6%)	23 (0.6%)
Unknown	1858 (0.6%)	10 (0.2%)
Marital status		
Unmarried ^a^	123,349 (39.6%)	2011 (49.4%)
Married	171,728 (55.1%)	1827 (44.9%)
Unknown	16,496 (5.3%)	229 (5.6%)
Insurance status		
Uninsured ^b^	5417 (1.7%)	163 (4.0%)
Insured	300,400 (96.4%)	3821 (94.0%)
Unknown	5756 (1.8%)	83 (2.0%)
Histology		
Infiltrating duct carcinoma	234,958 (75.4%)	2971 (73.1%)
Lobular carcinoma	27,050 (8.7%)	246 (6.0%)
Infiltrating duct and lobular carcinoma	16,392 (5.3%)	133 (3.3%)
Other types ^c^	33,173 (10.6%)	717 (17.6%)
Pathological Grade		
I	66,365 (21.3%)	144 (3.5%)
II	129,786 (41.6%)	1147 (28.2%)
III/IV	98,850 (31.7%)	1939 (47.7%)
Unknown	16,572 (5.3%)	837 (20.6%)
Surgery of primary site		
Yes	285,989 (91.8%)	952 (23.4%)
No	22,831 (7.3%)	3052 (75.0%)
Unknown	2753 (0.9%)	63 (1.5%)
Radiotherapy		
Yes	162,703 (52.2%)	1082 (26.6%)
No/Unknown	148,870 (47.8)	2985 (73.4%)
Chemotherapy		
Yes	130,572 (42.0%)	2802 (68.9%)
No/Unknown	181,001 (58.1%)	1265 (31.1%)
Extrahepatic metastatic sites to lung, brain and bone, No		
0	298,407 (95.8%)	1060 (26.1%)
1	9408 (3.0%)	1696 (41.7%)
2	2747 (0.9%)	891 (21.9%)
All 3	343 (0.1%)	177 (4.4%)
Unknown	668 (0.2%)	243 (6.0%)
Subtype		
HR+/HER2−	211,127 (67.8%)	1612 (39.6%)
HR+/HER2+	32,962 (10.6%)	884 (21.7%)
HR−/HER2+	14,089 (4.5%)	601 (14.8%)
Triple-negative	33,352 (10.7%)	544 (13.4%)
Unknown	20,043 (6.4%)	426 (10.5%)

Notes: ^a^ including divorced, separated, single (never married), and widowed; ^b^ including insured, Insured/No specifics Any Medicaid; ^c^ including other histology of invasive breast cancer except Infiltrating duct carcinoma, Lobular carcinoma and Infiltrating duct and lobular carcinoma; + denotes positive; − denotes negative; * denotes a statistically significant *P*-value; *HER2* Human epidermal growth factor receptor 2, *HR* Hormone receptor, *OR* Odds ratio, *CI* Confidence interval

**Table 2:** Baseline characteristics of breast cancer patients with liver metastases in the FUSCC dataset

Variable	Patients, No. %
	Patients (*n* = 1728)
Age at diagnosis, y	
21–40	288 (16.7%)
41–60	1143 (66.1%)
> 60	297 (17.2%)
Histology	
Infiltrating duct carcinoma	1396 (80.8%)
Lobular carcinoma	31 (1.8%)
Other	301 (17.4%)
De novo metastatic diseases	
No	1526 (88.3%)
Yes	202 (11.7%)
Surgery of primary site	
No	219 (12.7%)
Yes	1509 (87.3%)
Prior Chemotherapy	
No	226 (13.1%)
Yes	1502 (86.9%)
Prior Radiotherapy	
No	848 (49.1%)
Yes	785 (45.4%)
Unknown	95 (5.5%)
Recurrent sequence	
First liver metastases	1087 (62.9%)
Subsequent liver metastases	641 (37.1%)
Extrahepatic metastatic sites to lung, brain, bone and lymph nodes, No	
0	561 (32.5%)
1	534 (30.9%)
2	382 (22.1%)
3	223 (12.9%)
All 4	28 (1.6%)
Subtype	
HR+/HER2−	767 (44.3%)
HR+/HER2+	305 (17.7%)
HR−/HER2+	321 (18.6%)
Triple-negative	270 (15.6%)
Unknown	65 (3.8%)

Notes: + denotes positive; − denotes negative; *denotes a statistically significant *P*-value; HER2, human epidermal growth factor receptor 2; HR, hormone receptor

As presented in Table S1, the 4067 BCLM patients accounted for 1.31% of the entire cohort and 25.6% of the MBC patients, including 1612 patients with HR+/HER2- subtype tumors (39.64%), 884 with HR+/HER2+ tumors (21.74%), 601 with HR−/HER2+ tumors (14.78%), 544 with triple-negative tumors (13.38%), and 426 with unknown tumors (10.47%). The proportion of patients with HR−/HER2+ tumors ranked highest (4.27% of the entire cohort and 44.13% of the metastatic subclass), while those with HR+/HER2- tumors ranked lowest proportion in both entire and metastatic patients (0.76% of the entire cohort and 19.34% of the metastatic subclass, Table S1). For patients from FUSCC, patients with first liver metastases attributed to 35.59% proportion of all MBC patients (Table S2).

### Risk factors for liver metastasis

Using breast cancer patients aged between 18 and 40 as reference, the increase of age was associated with significant trend towards decreased risk of liver metastasis, with OR of 0.59, 0.46, and 0.46 for those aged between 41 and 60, 61–80 and those older than 80-years age, respectively (*P* < 0.001 for all, Table 3). The risk of liver metastasis was decreased in Hispanic (OR = 0.82, 95% CI = 0.74–0.91; *P* < 0.001) and Asian or Pacific Islander patients (OR = 0.81; 95% CI = 0.72–0.92; *P* = 0.001), but was increased for black patients (OR = 1.54; 95% CI = 1.41–1.67; *P* < 0.001) in comparison with white patients. Married (OR = 0.64; 95% CI = 0.60–0.68; *P* < 0.001) and insured status (OR = 0.53; 95% CI = 0.45–0.63; *P* < 0.001) was associated with significantly decreased risk of liver metastasis when compared with the status of unmarried and uninsured, respectively. Compared with infiltrating duct carcinoma, lobular carcinoma (OR = 0.68; 95% CI = 0.59–0.77; *P* < 0.001) and the mix of infiltrating duct and lobular carcinoma (OR = 0.66; 95% CI = 0.55–0.79; *P* < 0.001) were both associated with decreased risk of liver metastasis. Tumors with higher pathological grade more inclined to metastasize to liver, in the comparison of grade II versus I (OR = 3.89; 95% CI = 3.28–4.65; *P* < 0.001) and grade III/IV versus I (OR = 8.59; 95% CI = 7.26–10.23; *P* < 0.001). The analysis on molecular subtype indicated that HR+/HER2+ (OR = 3.13; 95% CI = 2.87–3.41; *P* < 0.001), HR−/HER2+ (OR = 4.75; 95% CI = 4.30–5.25; *P* < 0.001), and triple-negative subtypes (OR = 1.92; 95% CI = 1.73–2.12; *P* < 0.001) were all predictors for increased risk of liver metastasis in comparison with HR+/HER2- subtype, indicating the important role in disease progression played by HER2 status.

**Table 3:** Multivariate Logistic Regression for the risk factors of liver metastases at initial diagnosis of breast Cancer in the SEER database

Variable	Among Entire Cohort	
	Multivariate	
	OR (95% CI)	*P* Value
Age at diagnosis, y		
18–40	1[Reference]	
41–60	0.59 (0.54–0.66)	< 0.001*
61–80	0.46 (0.41–0.51)	< 0.001*
> 80	0.46 (0.40–0.54)	< 0.001*
Race		
White	1[Reference]	
Black	1.54 (1.41–1.67)	< 0.001*
Hispanic	0.82 (0.74–0.91)	< 0.001*
Asian or Pacific Islander	0.81 (0.72–0.92)	0.001*
American Indian/Alaska Native	0.93 (0.59–1.38)	0.725
Unknown	0.38 (0.19–0.68)	< 0.001*
Marital status		
Unmarried ^a^	1[Reference]	
Married	0.64 (0.60–0.68)	< 0.001*
Unknown	0.83 (0.72–0.96)	0.013*
Insurance status		
Uninsured ^b^	1[Reference]	
Insured	0.53 (0.45–0.63)	< 0.001*
Unknown	0.48 (0.35–0.67)	< 0.001*
Histology		
Infiltrating duct carcinoma	1[Reference]	
Lobular carcinoma	0.68 (0.59–0.77)	< 0.001*
Infiltrating duct and lobular carcinoma	0.66 (0.55–0.79)	< 0.001*
Other types ^c^	1.25 (1.14–1.37)	< 0.001*
Pathological Grade		
I	1[Reference]	
II	3.89 (3.28–4.65)	< 0.001*
III/IV	8.59 (7.26–10.23)	< 0.001*
Unknown	20.75 (17.32–25.02)	< 0.001*
Extrahepatic metastatic sites to lung, brain and bone, No		
0	1[Reference]	
1	58.49 (53.84–63.57)	< 0.001*
2	123.15 (110.89–136.76)	< 0.001*
All 3	246.17 (195.34–310.33)	< 0.001*
Unknown	299.10 (240.01–372.94)	< 0.001*
Subtype		
HR+/HER2−	1[Reference]	
HR+/HER2+	3.13 (2.87–3.41)	< 0.001*
HR−/HER2+	4.75 (4.30–5.25)	< 0.001*
Triple-negative	1.92 (1.73–2.12)	< 0.001*
Unknown	1.48 (1.31–1.68)	< 0.001*

Notes: ^a^ including divorced, separated, single (never married), and widowed; ^b^ including insured, Insured/No specifics Any Medicaid; ^c^ including other histology of invasive breast cancer except Infiltrating duct carcinoma, Lobular carcinoma and Infiltrating duct and lobular carcinoma; + denotes positive; − denotes negative; * denotes a statistically significant *P*-value; *HER2* Human epidermal growth factor receptor 2, *HR* Hormone receptor, *OR* Odds ratio, *CI* Confidence interval

### Survival and prognostic factors

**Fig. 1:**
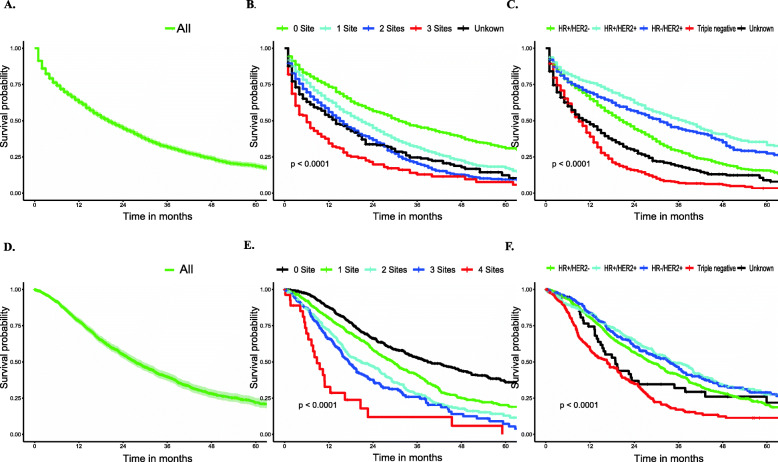
Overall Survival Among Patients of Breast Cancer with Liver Metastases. **a** Overall survival (SEER). **b** Survival stratified by the extent of extrahepatic metastatic disease (SEER). **c** Survival stratified by subtype (SEER). **d** Overall survival (FUSCC). **e** Survival stratified by the extent of extrahepatic metastatic disease (FUSCC). **f** Survival stratified by subtype (FUSCC)

**Fig. 2:**
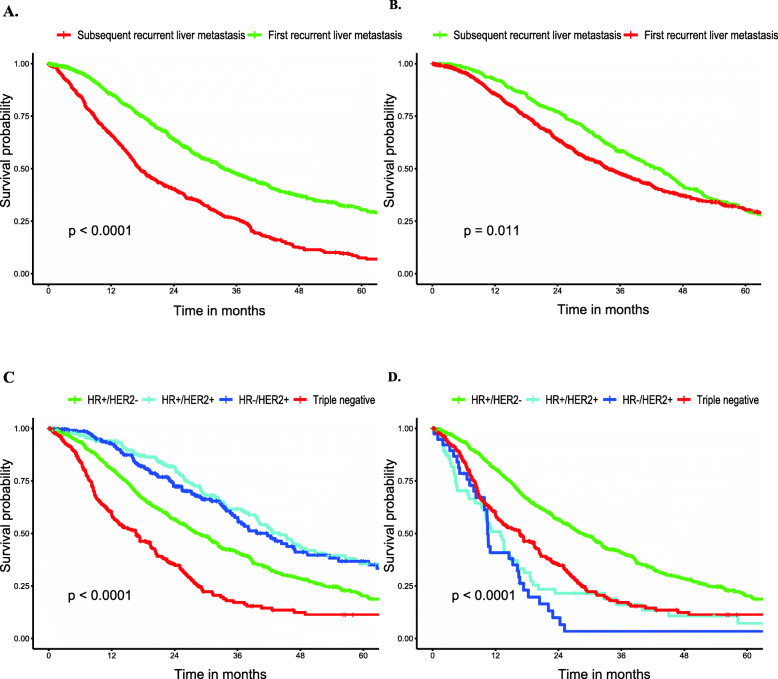
**a** Survival stratified by Recurrent sequence (Time from diagnosis of liver metastases). **b** Survival stratified by Recurrent sequence (Time from metastasis). **c** Overall Survival Among Patients of Breast Cancer Liver Metastases with targeted therapy stratified by subtype (FUSCC). **d** Overall Survival Among Patients of Breast Cancer Liver Metastases without targeted therapy stratified by subtype (FUSCC)

Median survival among BCLM patients, as stratified by subtype, is displayed in Tables S1 and S2. The median survival among the entire cohort was 20.00 months in the SEER database (vs 27.30 months in the FUSCC dataset), with patients with the HR+/HER2+ subtype experiencing the longest median survival (38.00 vs 34.00 months) and patients with the triple-negative subtype experiencing the shortest median survival (9.00 vs 15.63 months) in the two cohorts. Additionally, breast cancer patients with first liver metastases showed distinctly longer survival times than those patients with subsequent liver metastases when the time was calculated from the diagnosis of liver metastasis (33.80 vs 17.47 months, Fig. 2a). However, patients with liver metastases had a shorter survival time than breast cancer patients developing liver metastases during the subsequent disease course when the time was calculated from the diagnosis of MBC (42.57 vs 33.80 months, Fig. 2b). The overall survival of all BCLM patients and the overall survival stratified by subtype or extent of extrahepatic metastatic disease are graphically displayed in Fig. 1.

Multivariate Cox proportional hazards models were used to assess the prognostic factors of patients with BCLM in the SEER database (Table 4) and the FUSSCC dataset was used for further exploration (Table 5). In the SEER database, older patients had worse survival, with a HR of 1.39, 1.84 and 3.62 for patients aged 41–60, 61–80, and > 80 years in comparison with those aged 18–40 years (*P* < 0.001 for all). Moreover, black and Hispanic race were associated with increased death risk compared with white race, with a HR of 1.35 (*P* < 0.001) and 1.16 (*P* = 0.028), respectively). Results also showed a prolonged survival in presence of the status of married and insurance (HR = 0.84 for married vs. unmarred, and 0.71 for insured vs. uninsured, with *P* < 0.001 for all). For the survival comparisons among clinical factors, we found that increased pathological grade, treatment without chemotherapy and surgery of primary site, increased number of extrahepatic metastatic sites and triple-negative pathological type were all associated with poor prognosis. Specifically, compared with grade I disease, the HR was 1.35 (*P* = 0.013) for grade II and 1.69 for grade III-IV (*P* < 0.001), respectively. Treatment without surgery of primary site and chemotherapy generated a HR of 1.52 and 1.63, respectively compared with those received the treatments (*P* < 0.001 for all). As expected, the HR increased from 1.42 to 3.43 as number of extrahepatic sites increased from 1 to 3, compared with no extrahepatic metastasis (*P* < 0.001 for all). In line of most previous studies, triple-negative subtype remained the deadliest type of cancer, with HR of 2.46 in comparison with HR+/HER2- cancers (*P* < 0.001). Interestingly, compared with HR+/HER2- subtype, we observed that HER2+ might decrease the death risk in the SEER database, with HR of 0.69 for HR+/HER2+ (*P* < 0.001) and 0.85 (*P* = 0.014) for HR−/HER2+ subtype, probably due to the introduction of HER2-targeted therapy.

**Table 4:** Univariate and Multivariate Cox Regression for OS of breast cancer with liver metastases at diagnosis in the SEER database

Variable	Overall Survival			
	Univariable		Multivariable	
	HR (95% CI)	*P* Value	HR (95% CI)	*P* Value
Age, y				
18–40	1[Reference]		1[Reference]	
41–60	1.52 (1.32–1.76)	< 0.001*	1.39 (1.20–1.61)	< 0.001*
61–80	2.17 (1.88–2.51)	< 0.001*	1.84 (1.58–2.15)	< 0.001*
> 80	4.44 (3.65–5.39)	< 0.001*	3.62 (2.76–4.74)	< 0.001*
Race ^a^				
White	1[Reference]		1[Reference]	
Black	1.21 (1.09–1.33)	< 0.001*	1.35 (1.22–1.50)	< 0.001*
Hispanic	0.99 (0.87–1.13)	0.895	1.16 (1.02–1.32)	0.028*
Asian or Pacific Islander	0.86 (0.74–1.02)	0.078	0.90 (0.76–1.05)	0.182
American Indian or Alaska Native	0.96 (0.58–1.59)	0.869	1.01 (0.60–1.68)	0.984
Unknown	0.34 (0.09–1.37)	0.129	0.28 (0.07–1.15)	0.078
Marital status				
Unmarried ^a^	1[Reference]		1[Reference]	
Married	0.75 (0.70–0.82)	< 0.001*	0.84 (0.77–0.91)	< 0.001*
Unknown	0.96 (0.81–1.14)	0.664	0.89 (0.75–1.06)	0.200
Insurance status				
Uninsured ^b^	1[Reference]		1[Reference]	
Insured	0.75 (0.62–0.91)	0.003*	0.71 (0.59–0.86)	< 0.001*
Unknown	0.94 (0.68–1.30)	0.717	0.83 (0.57–1.22)	0.348
Histology				
Infiltrating duct carcinoma	1[Reference]		1[Reference]	
Lobular carcinoma	1.14 (0.97–1.34)	0.112	0.87 (0.74–1.03)	0.101
Infiltrating duct and lobular carcinoma	0.94 (0.68–1.30)	0.717	0.89 (0.70–1.13)	0.323
Other types ^c^	1.43 (1.30–1.58)	< 0.001*	1.18 (1.05–1.33)	0.005*
Pathological Grade				
I	1[Reference]		1[Reference]	
II	1.08 (0.86–1.37)	0.515	1.35 (1.07–1.72)	0.013*
III/IV	1.30 (1.04–1.64)	0.022*	1.69 (1.34–2.14)	< 0.001*
Unknown	1.59 (1.26–1.01)	< 0.001*	1.59 (1.23–2.05)	< 0.001*
Surgery of primary site				
Yes	1[Reference]		1[Reference]	
No	1.72 (1.56–1.89)	< 0.001*	1.52 (1.38–1.68)	< 0.001*
Unknown	0.93 (0.62–1.38)	< 0.001*	0.96 (0.64–1.43)	< 0.001*
Radiotherapy				
Yes	1[Reference]		1[Reference]	
No/Unknown	1.03 (0.94–1.12)	0.528	0.95 (0.87–1.04)	0.270
Chemotherapy				
Yes	1[Reference]		1[Reference]	
No/Unknown	2.03 (1.87–2.20)	< 0.001*	1.63 (1.50–1.78)	< 0.001*
Extrahepatic metastatic sites to lung, brain and bone, No				
0	1[Reference]		1[Reference]	
1	1.49 (1.34–1.65)	< 0.001*	1.42 (1.28–1.58)	< 0.001*
2	1.94 (1.73–2.18)	< 0.001*	1.85 (1.65–2.09)	< 0.001*
All 3	2.94 (2.44–3.56)	< 0.001*	3.43 (2.77–4.26)	< 0.001*
Unknown	1.92 (1.62–2.28)	< 0.001*	1.62 (1.36–1.93)	< 0.001*
Subtype				
HR+/HER2−	1[Reference]		1[Reference]	
HR+/HER2+	0.59 (0.53–0.66)	< 0.001*	0.69 (0.61–0.77)	< 0.001*
HR−/HER2+	0.72 (0.63–0.82)	< 0.001*	0.85 (0.74–0.97)	0.014*
Triple-negative	2.10 (1.88–2.35)	< 0.001*	2.46 (2.18–2.77)	< 0.001*
Unknown	1.54 (1.36–1.74)	< 0.001*	1.38 (1.20–1.58)	< 0.001*

Notes: ^a^ including divorced, separated, single (never married), and widowed; ^b^ including insured, Insured/No specifics Any Medicaid; ^c^ including other histology of invasive breast cancer except Infiltrating duct carcinoma, Lobular carcinoma and Infiltrating duct and lobular carcinoma;

+ denotes positive; − denotes negative; * denotes a statistically significant *P*-value; *HER2* Human epidermal growth factor receptor 2, *HR* Hormone receptor, *OR* Odds ratio, *CI* Confidence interval

**Table 5:** Univariate and Multivariate Cox Regression for OS among patients of Breast Cancer with Liver Metastases in the FUSCC dataset

Variable	Overall Survival			
	Univariate		Multivariate	
	HR (95% CI)	*P* Value	HR (95% CI)	*P* Value
Age at diagnosis, y				
21–40	1[Reference]		1[Reference]	
41–60	1.34 (1.11–1.62)	0.003*	1.38 (1.09–1.74)	0.006*
> 60	1.39 (1.10–1.75)	0.005*	1.55 (0.86–2.80)	0.145
Histology				
Infiltrating duct carcinoma	1[Reference]		1[Reference]	
Lobular carcinoma	0.90 (0.56–1.43)	0.648	0.68 (0.41–1.14)	0.146
Other	0.72 (0.60–0.87)	< 0.001*	1.05 (0.85–1.29)	0.661
De novo metastatic diseases				
No	1[Reference]		1[Reference]	
Yes	0.40 (0.31–0.52)	< 0.001*	0.67 (0.29–1.52)	0.337
Surgery of primary site				
No surgery	1[Reference]		1[Reference]	
Yes	2.33 (1.83–2.98)	< 0.001*	1.02 (0.50–2.09)	0.947
Prior Chemotherapy				
No	1[Reference]		1[Reference]	
Yes	2.56 (1.99–3.28)	< 0.001*	1.51 (0.88–2.60)	0.135
Prior Radiotherapy				
No	1[Reference]		1[Reference]	
Yes	1.33 (1.17–1.51)	< 0.001*	1.20 (1.04–1.39)	0.014*
Unknown	0.77 (0.54–1.08)	0.122	0.67 (0.45–0.99)	0.046*
Recurrent sequence				
First liver metastases	1[Reference]		1[Reference]	
Subsequent liver metastases	2.16 (1.90–2.46)	< 0.001*	1.58 (1.33–1.87)	< 0.001*
Extrahepatic metastatic sites to lung, brain, bone and lymph nodes, No				
0	1[Reference]		1[Reference]	
1	1.54 (1.30–1.83)	< 0.001*	1.16 (0.95–1.41)	0.15
2	2.16 (1.80–2.59)	< 0.001*	1.56 (1.23–1.97)	< 0.001*
3	2.57 (2.08–3.18)	< 0.001*	2.05 (1.51–2.79)	< 0.001*
All 4	5.49 (3.56–8.47)	< 0.001*	4.59 (2.42–8.69)	< 0.001*
Subtype				
HR+/HER2−	1[Reference]		1[Reference]	
HR+/HER2+	0.82 (0.68–0.98)	0.033*	1.02 (0.83–1.25)	0.851
HR−/HER2+	0.84 (0.70–1.01)	0.067	1.13 (0.92–1.40)	0.242
Triple-negative	1.83 (1.53–2.20)	< 0.001*	2.17 (1.78–2.63)	< 0.001*
Unknown	1.11 (0.79–1.57)	0.544	1.63 (1.10–2.43)	0.015*

Notes: + denotes positive; − denotes negative; *denotes a statistically significant *P*-value; HER2, human epidermal growth factor receptor 2; HR, hormone receptor; CI confidence interval

Despite the substantial differences in baseline characteristics, the significant association of older age and greater number of extrahepatic metastasis sites with worse prognosis of the BCLM patients was also successfully observed in FUSCC datasets, similar to the former observation in the SEER database (Table 5). Additionally, histological type exerted no effect on the prognosis and patients with triple negative BCLM had the worst survival in both SEER and FUSCC dataset. Notably, specific results were obtained due to data availability in two different populations, such as race, marital and insurance status, pathological grade and recurrent sequence. However, no significant difference was observed between survival of HER2+ patients with HR+/HER2- patients (*P* > 0.05) in FUSCC dataset different from the result of SEER database, probably owing to difference in clinical application of HE2-targeted therapies.

### HER2-targeted therapy

Owing to inconsistent results in the terms of the prognostic influence of molecular subtype in BCLM patients, we next explored whether HER2-targeted therapy leaded to these results. According to the results from the FUSCC, we found that in patients who did not receive HER-2 targeted therapy after liver metastases, HER2+ patients had an unfavorable prognosis compared with HR+/HER2- patients, with HR of 2.62 for HR+/HER2+ and 3.43 for HR−/HER2+ patients (*P* < 0.001 for all, Table 6). However, HR+/HER2+ patients had a better prognosis than HR+/HER2- patients in patients who underwent HER2-targeted therapy after liver metastasis, with HR of 0.74 (*P* < 0.001). Unfortunately, we only observed an insignificant trend towards decreased death risk induced by HER2-targeted therapy for HR−/HER2+ patients compared with HR+/HER2- patients, with a HR of 0.81 (*P* = 0.110). Overall survival among BCLM patients with or without HER2-targeted therapy stratified by subtype were visualized in Fig. 2.

**Table 6:** Multivariate Cox Regression for OS among patients of Breast Cancer Liver Metastases with or without HER2-targeted therapy in the FUSCC dataset

Variable	Overall Survival With HER2-targeted therapy		Overall Survival Without HER2-targeted therapy	
	HR (95% CI)	*P* Value	HR (95% CI)	*P* Value
Subtype				
HR+/HER2−	1[Reference]		1[Reference]	
HR+/HER2+	0.74 (0.58–0.95)	0.017*	2.62 (1.88–3.66)	< 0.001*
HR−/HER2+	0.81 (0.63–1.05)	0.110	3.43 (2.29–5.15)	< 0.001*
Triple-negative	2.17 (1.78–2.63)	< 0.001*	2.17 (1.78–2.63)	< 0.001*

Adjust for other variables

+ denotes positive; − denotes negative; * denotes a statistically significant *P*-value; *HER2* Human epidermal growth factor receptor 2, *HR* Hormone receptor, *CI* Confidence interval

## Discussion

The risk factors for liver metastasis and prognostic factors of BCLM patients was described in this study. It was reported that the incidence of liver as the first metastatic site varied from 17.8–35% [7, 19, 20]. We found that the incidence of first liver metastases was 25.6% in the SEER database and 35.59% in the FUSCC database, similar to these previous studies.

In addition to multiple molecular mechanisms underlying liver metastasis in breast cancer patients, a positive correlation between the occurrence of liver metastasis and multiple clinicopathological features, such as young age, invasive ductal carcinoma, higher pathological grade, and subtype of HER2+, was found in our study. The risk for liver metastasis surprisingly decreased with increasing age, which may result from a decrease in tumor-proliferative factors or deterioration of the immune system among patients of advanced age [21, 22]. Zengel et al. found that invasive lobular carcinoma and mixed-type tumors had a higher incidence of bone metastasis than invasive ductal carcinoma, suggesting the potential role of histological type in the development of liver metastasis of breast cancer [23]. Infiltrating duct carcinoma was also found to be strongly associated with the occurrence of liver metastasis in our study. Purushotham et al. demonstrated that the higher the histological grade of the breast cancer, the higher the risk of developing visceral metastasis because high histological grade also correlated positively with proliferation and metastasis capacity of tumor cells [22]. In addition, our present results also confirmed that HER2-positive or triple-negative subtype had significantly greater odds of developing liver metastases than HR+/HER2- subtype, consistent with previously published studies focused on the metastatic pattern of different breast cancer subtype [10, 19, 20, 22]. HER2 could upregulate the expression of the chemokine receptor CXCR4 and therefore promoted liver metastasis via the CXCL12/CXCR4 pathway, while elevated expression of the fibroblast growth factor homologous factor (FGF13) could mediate the formation of liver metastases in the triple negative breast cancer, resulting in molecular subtype-based liver metastasis of breast cancer [24–26]. A recent study reported the first discovery of the functional role of the DNA of neutrophil extracellular traps (NETs) in promoting breast cancer liver metastasis, a mechanism distinct from previous studies on cytokines and chemokine receptors, integrin complexes, metabolic program and proliferation signaling [27, 28]. Early detection of liver metastases seemed to confer better outcomes of specific patients due to more effective treatments and better tolerance [29–34]. However, guidelines have reiterated that routine laboratory and imaging examination may not be applicable for patients with early breast cancer in the absence of signs or symptoms of metastatic disease because these examinations may not bring more survival benefits [35–37]. Our results can help to identify breast cancer patients at high risk for developing liver metastasis.

The median survival after liver metastasis was 20.00 months in the SEER database (vs 27.30 months in the FUSCC dataset) and varied significantly by molecular subtype. In both SEER database and FUSCC dataset, HR+/HER2+ patients had the longest survival (38.00 vs 34.00 months), whereas triple negative breast cancer had the worst prognosis (9.00 vs 15.63 months). Both HR+/HER2+ and HR−/HER2 + BCLM patients had a more favorable outcome than HR+/HER2-BCLM patients in the SEER database, but there was no significant difference in the FUSCC dataset. We hypothesized that it may originate from disparities in the application of HER2-targeted therapy. According to the results from FUSCC, HER2 positive BCLM patients who did not receive HER2-targeted therapy after liver metastases had worse outcomes than HR+/HER2- patients, whereas significantly improved clinical outcomes were observed among those patients undergoing HER2-targeted therapy, in accordance with results from the SEER database. While there was still not a substantial survival advantage of HR+/HER2- BCLM patients receiving HER2-targeted therapy in the FUSCC dataset, our findings could indicate that HR+/HER2+ or even HR−/HER2+ BCLM patients had a favorable prognosis than HR+/HER2- BCLM patients owing to the introduction of HER2-targeted therapy. The gene that encodes HER2 is amplified and overexpressed in 15–20% of newly diagnosed breast cancer and results in a worse survival [38, 39]. Nevertheless, diverse HER2-directed drugs have significantly improved survival in breast cancer patients with HER2-positive subtype [40–45]. Even for HER2­positive metastatic breast cancer, anti­HER2 therapy also results in considerable and long-lasting improvement in quality of life and overall survival [46]. Furthermore, continuous anti-HER2 therapy is of utmost significance for the improvement of survival outcomes in metastatic breast cancer [42, 47, 48]. However, these treatments are expensive and require professional guidance from oncologists, limiting their availability and continuous use for patients without health insurance or in lower income countries, which may explain differences in outcome between these two populations [46]. These inconsistent results on the prognostic role of molecular subtype in BCLM patients may also be explained by differences in baseline characteristics of two groups.

Novel treatment options and different metastatic sites profoundly changed the prognostic value of molecular subtype in breast cancer patients. HR+/HER2- breast cancer were historically considered to have a relatively favorable prognosis, whereas HR+/HER2+ subtype seemed to have the best prognosis and HR+/HER2- and HR−/HER2+ subtype had similar survival among patients with de novo metastatic breast cancer in the HER2-targeted therapy era [49, 50]. Among breast cancer patients with de novo brain or bone metastases, results were consistent with those in de novo metastatic breast cancer patients [51, 52], while HR−/HER2+ subtype had a worse prognosis than the HR+/HER2- subtype among breast cancer patients with de novo lung metastases [53]. Differently, HR−/HER2+ BCLM patients may have a better prognosis than the HR+/HER2- subtype in our study. There are many potential reasons for these discrepancies. HR+/HER2- patients with visceral metastases are often considered to insensitive to endocrine therapy than those without visceral metastases [54]. In the FALCON study, the median progression-free survival (PFS) in patients receiving fulvestrant 500 mg as first-line treatment with and without visceral disease was 13.8 months and 22.3 months, respectively [55]. M. He et al. found that heterogeneity existed among different visceral metastatic sites, and the median PFS was longer in patients with lung metastases than in those with liver metastases after fulvestrant therapy (9.6 and 3.7 months, respectively, *P* < 0.001) [54]. Kimbung et al. also identified a 17-gene liver metastasis-specific signature, which was significantly and independently prognostic for poor relapse-free and overall survival in ER-positive tumors [56]. Fortunately, substantial progress has been made after major advances in our understanding of the biology of ER+/HER2– breast cancer in the past 20 years, such as the development of CDK4/6 inhibitors, mTOR inhibitors, PI3Kα inhibitors and histone deacetylase inhibitors [57]. Subgroup analyses suggested that patients with visceral metastases also benefited from the addition of these targeted therapies to endocrine therapy [58–61]. On the basis of the above findings, endocrine therapy combined with these novel targeted therapies may be more appropriate for HR+/HER2- BCLM patients than endocrine therapy alone, but further research is required.

## Limitations

We acknowledge that there are some limitations in our study. First, it was retrospective study including two groups with different baseline characteristics, yielding relatively different results. Second, some detailed information that may have effects on survival was not available, including other metastatic sites, such as the pleura and contralateral breast, number and maximum diameter of liver metastases and performance status. Third, we did not evaluate whether different HER2-targeted therapies, endocrine therapies and surgery on the prognosis of BCLM patients to provide individualized treatment for these specific populations.

## Conclusions

Despite these limitations, our study had a larger sample size and the majority of patients were more recently diagnosed than previous studies on this subject. As a result, our findings may be more reliable and representative. Breast cancer patients with young age, invasive ductal carcinoma, higher pathological grade, and triple-negative and HER2+ subtypes have a high risk of developing liver metastases at initial diagnosis, and therefore deserve more attention during the follow-up. Furthermore, HR+/HER2+ or even HR−/HER2+ BCLM patients had a better prognosis than HR+/HER2- BCLM patients owing to the introduction of HER2-targeted therapy. HER2-targeted based therapy may be the cornerstone of treatment for HER2+ BCLM patients.

## Supplementary Information

**Additional file 1.** **Figure S1**. Selection of patients (SEER).

**Additional file 2.** **Figure S2**. Selection of patients (FUSCC).

## Data Availability

The datasets of the present study were available from the corresponding author on reasonable request.

## Abbreviations

OS: Overall survival
BCLM: Breast cancer with liver metastases
SEER: Surveillance, Epidemiology, and End Results
FUSCC: Fudan University Shanghai Cancer Center
HER2: Human epidermal growth factor receptor 2 positive
+: positive
HR: Hormone receptor
HR: Hazard ratio
MBC: Metastatic breast cancer
ER: Estrogen receptor
LM: Liver metastases
IQR: Interquartile range
-: Negative
ORs: Odds ratios
CIs: Confidence intervals
FGF13: Fibroblast growth factor homologous factor
NETs: Neutrophil extracellular traps
PFS: Progression-free survival
