# Supplementary material for: Risk and prognostic factors of breast cancer with liver metastases
Source: BMC Cancer. 2021 Mar 6;21:238. doi: 10.1186/s12885-021-07968-5 (PMC7937288; doi:10.1186/s12885-021-07968-5)
Supplement: Supplementary file 1 — Additional file 1. Figure S1. Selection of patients (SEER). [file 12885_2021_7968_MOESM1_ESM.zip › Table S1R3.docx]

| Table S1. Incidence Proportion and Median Survival of Patients With Breast Cancer With Identified  Liver Metastases at Diagnosis by Subtype (**SEER**) | | | | | | |
| --- | --- | --- | --- | --- | --- | --- |
| **Subtype** | **Patients, No. (%)** | | | **Incidence Proportion of Liver Metastases, %** | | **Survival Among Patients With Liver Metastases, Median, months** |
|  | With Breast  Cancer | With Metastatic  Disease | With Liver  Metastases | Among Entire Cohort | Among Subset With Metastatic Disease |  |
| HR+/HER2- | 211127(67.76) | 8337(52.49) | 1612(39.64) | 0.76 | 19.34 | 21.00 |
| HR+/HER2+ | 32962(10.58) | 2511(15.81) | 884(21.74) | 2.68 | 35.21 | 38.00 |
| HR-/HER2+ | 14089(4.52) | 1362(8.57) | 601(14.78) | 4.27 | 44.13 | 31.00 |
| Triple-negative | 33352(10.70) | 1988(12.52) | 544(13.38) | 1.63 | 27.36 | 9.00 |
| Unknown | 20043(6.43) | 1686(10.61) | 426(10.47) | 2.13 | 25.27 | 11.00 |
| All subtypes | 311573(100) | 15884(100) | 4067(100) | 1.31 | 25.60 | 20.00 |

NOTE. HER2, human epidermal growth factor receptor 2; HR, hormone receptor; NA, Not Available. + Denotes positive; - denotes negative;
