# Supplementary figures and images for: Risk and prognostic factors of breast cancer with liver metastases
Source: BMC Cancer. 2021 Mar 6;21:238. doi: 10.1186/s12885-021-07968-5 (PMC7937288; doi:10.1186/s12885-021-07968-5)

A.

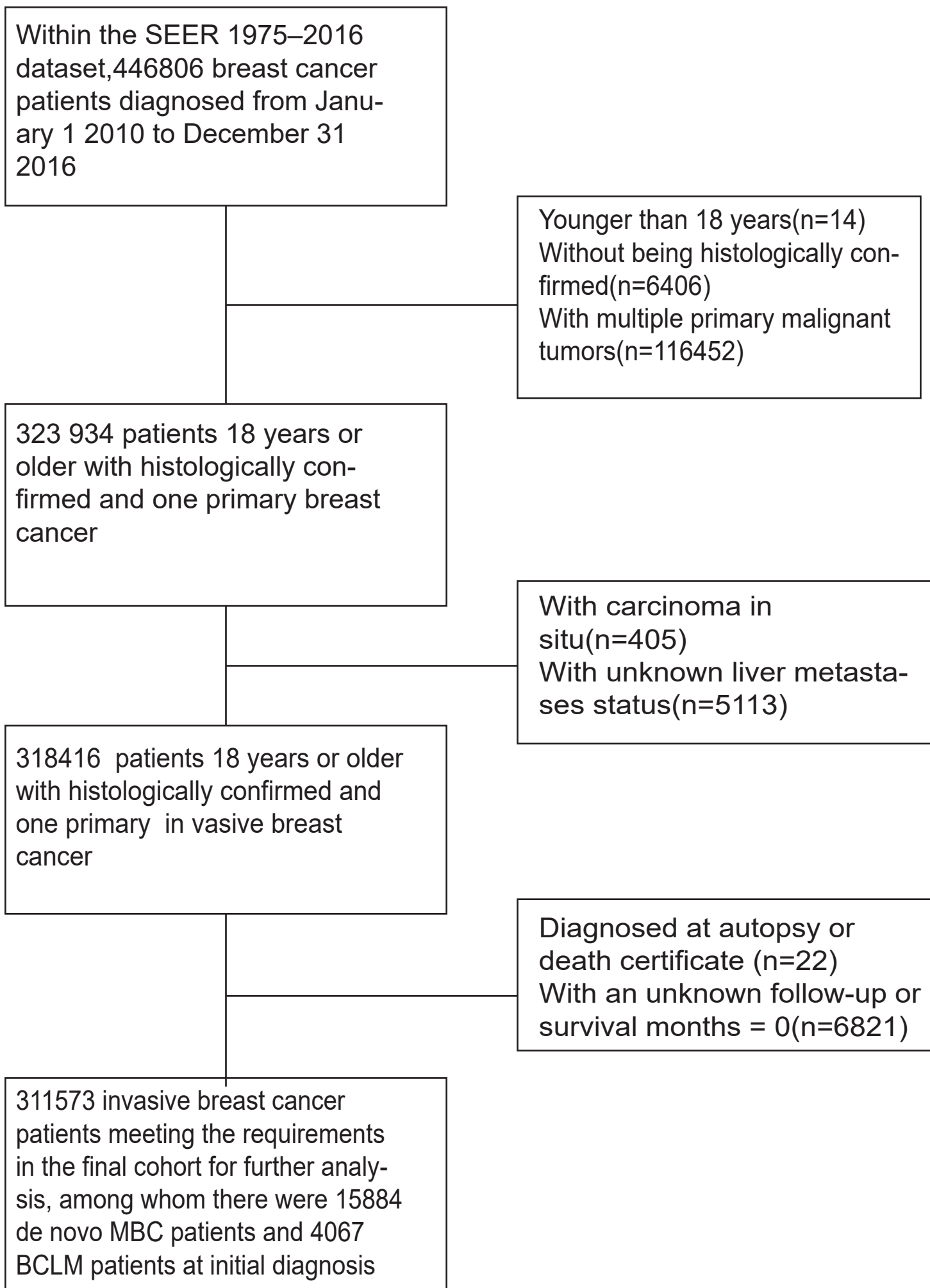

Supplement: Supplementary file 1 — Additional file 1. Figure S1. Selection of patients (SEER). [file 12885_2021_7968_MOESM1_ESM.zip › Figure S1R3.pdf]

B.

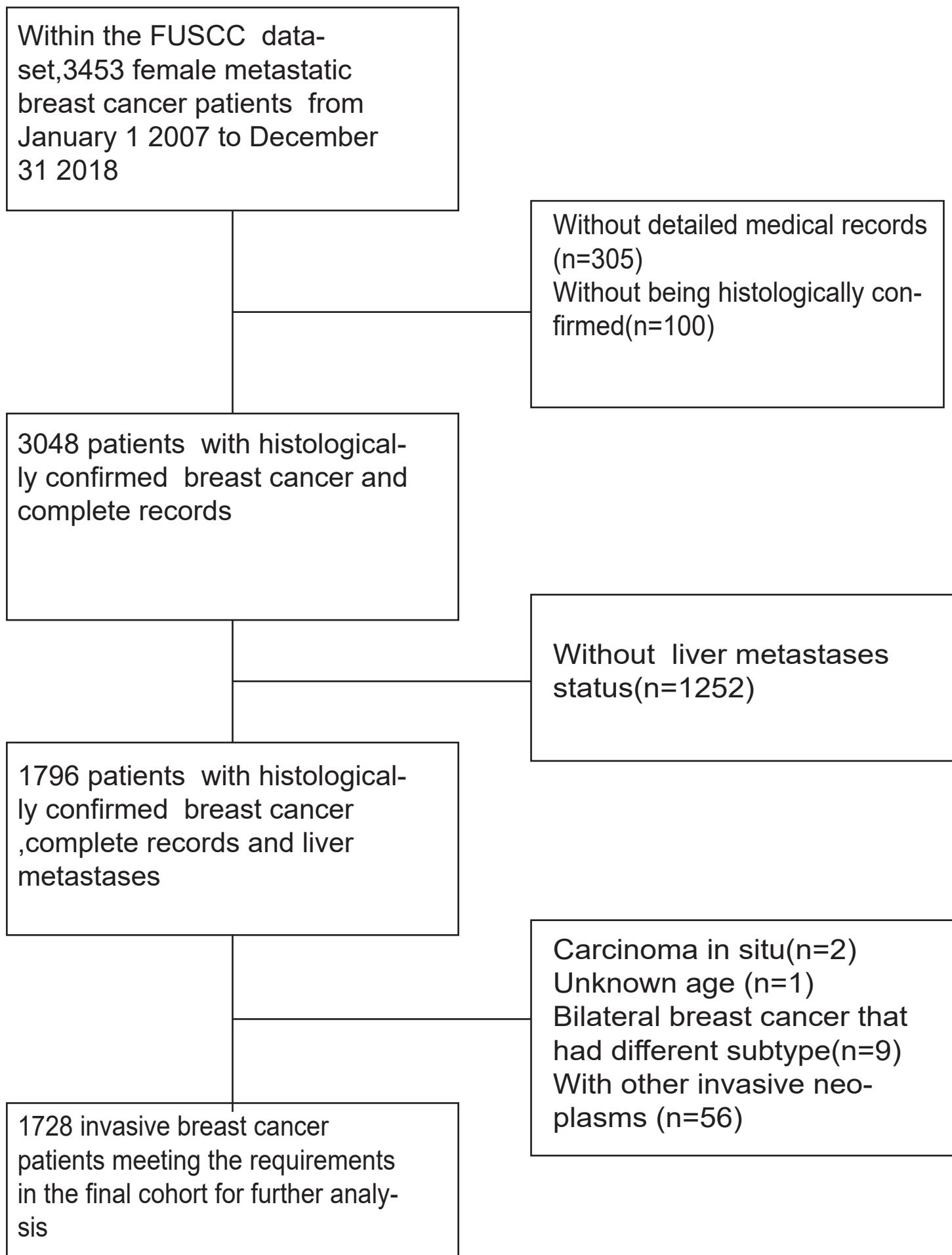

Supplement: Supplementary file 2 — Additional file 2. Figure S2. Selection of patients (FUSCC). [file 12885_2021_7968_MOESM2_ESM.zip › Figure S2R3.pdf]
