# Supplementary material for: Risk and prognostic factors of breast cancer with liver metastases
Source: BMC Cancer. 2021 Mar 6;21:238. doi: 10.1186/s12885-021-07968-5 (PMC7937288; doi:10.1186/s12885-021-07968-5)
Supplement: Supplementary file 2 — Additional file 2. Figure S2. Selection of patients (FUSCC). [file 12885_2021_7968_MOESM2_ESM.zip › Table S2R3.docx]

| Table 2. Proportion and Median Survival among Patients of Breast Cancer With Identified Liver Metastases by Subtype (**FUSCC**) | | | | | | |
| --- | --- | --- | --- | --- | --- | --- |
| Subtype | Patients of Liver Metastases, No. (%) | | | Survival ^a^ Among Patients With Liver Metastases, Median (95% CI), months | | |
|  | With Breast Cancer Liver Metastases | With First Liver  Metastases ^b^ | With Subsequent Liver Metastases ^C^ | Among Entire population | With First Liver  Metastases | With Subsequent Liver Metastases |
| HR+/HER2- | 767(44.39) | 414(38.09) | 353(55.07) | 28.53(26.10-31.80) | 38.30(32.30-44.17) | 18.47(16.93-23.27) |
| HR+/HER2+ | 305(17.65) | 215(19.78) | 90(14.04) | 34.00(29.40-40.30) | 42.43(38.17-58.50) | 20.87(16.30-29.30) |
| HR-/HER2+ | 321(18.58) | 249(22.91) | 72(11.23) | 33.60(27.33-38.03) | 35.47(29.93-42.37) | 18.77(13.77-45.40) |
| Triple-negative | 270(15.63) | 170(15.63) | 100(15.60) | 15.63(12.50-19.47) | 20.20(16.60-23.37) | 9.10(8.20-13.50) |
| Unknown | 65(3.76) | 39(3.59) | 26(4.06) | 18.90(14.87-34.87) | 23.30(14.17-NA) | 15.67(13.30-NA) |
| All subtypes | 1728(100) | 1087(100) | 641(100) | 27.30(25.57-29.40) | 33.80(31.20-37.73) | 17.47(16.30-19.77) |

NOTE. HER2, human epidermal growth factor receptor 2; HR, hormone receptor; NA, Not Available; + Denotes positive; − denotes negative; ^a^, Time from diagnosis of liver metastases; ^b^, including the presence of liver metastases at initial diagnosis or liver metastases as the first metastatic site; ^C^, including the presence of liver metastases during the subsequent clinical course; CI, confidence interval
